# Development of Electrochromic Devices, Based on Polymeric Gel, for Energy Saving Applications

**DOI:** 10.3390/polym15163347

**Published:** 2023-08-09

**Authors:** Carmen Rizzuto, Riccardo C. Barberi, Marco Castriota

**Affiliations:** 1 Department of Physics, University of Calabria Ponte Bucci, Cubo 33B, 87036 Rende, CS, Italy; 2 CNR-Nanotec c/o Department of Physics, University of Calabria Ponte Bucci, Cubo 33B, 87036 Rende, CS, Italy

**Keywords:** viologen, UV-Vis-NIR and Raman spectroscopies, cyclic voltammetry, color contrast ratio, coloration efficiency

## Abstract

In this work, the implementation of an electrochromic device (10 cm × 10 cm in size) for energy saving applications has been presented. As electrochromic system has been used with an electrochromic solution (ECsol) made by ethyl viologen diperchlorate [EV(ClO_4_)_2_], 1,1′-diethyl ferrocene (DEFc) and propylene carbonate (PC), as solvent. The final system has been obtained by mixing the ECsol, described above, with a polymeric system made by Bisphenol-A glycerolate (1 glycerol/phenol) diacrylate (BPA) and 2,2-Dimethoxy-2-phenylacetophenone (Irgacure 651) in a weight percentage equal to 60:40% *w*/*w*, respectively. Lithography has been used to make a spacer pattern with a thickness of about 15–20 µm between the two substrates. Micro-Raman spectroscopy confirmed the presence of the EV^•+^ as justified by the blue color of the electrochromic device in the ON state. Electrochemical and optical properties of the electrochromic device have been studied. The device shows reversible electrochromic behavior as confirmed by cyclic color variation due to the reduction and oxidation process of the EV^2+^/EV^•+^ couple. The electrochromic device shows a variation of the % transmittance in the visible region at 400 nm of 59.6% in the OFF state and 0.48% at 3.0 V. At 606 nm the transmittance in the bleached state is 84.58% in the OFF state and then decreases to 1.01% when it is fully colored at 3.0 V. In the NIR region at 890 nm, the device shows a transmittance of 74.3% in the OFF state and 23.7% at 3.0 V while at 1165 nm the values of the transmittance changed from 83.21% in the OFF state to 1.58% in the ON state at 3.0 V. The electrochromic device shows high values of CCR% and exhibits excellent values of CE in both visible and near-infrared regions when switched between OFF/ON states. In the NIR region at 890 nm, electrochromic devices can be used for the energy-saving of buildings with a promising CE of 120.9 cm^2^/C and 420.1 cm^2^/C at 1165 nm.

## 1. Introduction

The phenomenon of electrochromism is attracting much attention because of its variable applications starting from smart windows up to lower power display systems [1,2,3] using different kinds of available electrochromic materials: organic [4] and inorganic [5,6,7].

Organic electrochromic materials such as innovative conjugated polymers exhibit fast response time, contrast color, lower power consumption, and color versatility, and can be easily used in a wide range of multifunctional applications (flexible display, smart windows, camouflage, etc.) [8]. Moreover, these kinds of materials are sensitive to external stimuli, and this means that their properties can be modulated by light, electricity, solvent stimulation, etc. [9]. Recently, with the introduction of heteroatoms in the chain of the polymer, such as polythiophenes [10], and the use of the D-A-D method [11] it is possible to obtain greater control of the electrical properties of the materials improving electrochromic performances of the devices [12]. Due to its durability and low synthetic costs, Prussian blue offers fast transmittance switching properties with no memory effect that together with tungsten trioxide are used as electrochromic layers by in situ polymerization for the production of complementary electrochromic devices [13,14].

One interesting class of materials is the bipyridinium species based on N, N-diquaternization of 4,4′-bipyridine, also known as viologens [4,15]. Viologen species are a reducing material that exhibit a reversible redox reaction because of the one or two-electron reductions, and they have been studied for their visible color change associated with their reductions, high color contrast, switchable electrochromic properties, and for the construction of flexible electrochromic devices [16,17]. These materials have been used in many applications e.g., as electron mediators, electrochromic display components, relay compounds in energy storage systems, and so on [18]. The color of the viologens is related to the type of the substituents (alkyl, benzyl, and aryl) present in the nitrogen position [19]. The well-known 1,1′-ethyl viologen diperchlorate presents three redox states which are assigned to the formation of the di-cation (EV^2+^, colorless), radical cation (EV^•+^, blue), and neutral viologen species (EV, brown) with their low reduction potentials. Electrochromic devices have been made up of different configurations: using viologen as cathodic and ferrocene as anodic species obtaining shorter time responses in the switching process of the electrochromic device or in addition to ionic liquid for improving the electrochemical stability of the electrolyte in the device or coupled with polythiophene to make a tristate electrochromic device or together with CNTs n-type doped in sandwich structures composed of a thiol-terminated viologen as a layer immobilized on an electrode of gold nanoparticles acting as a plasmonic antenna; or combined with metal oxide-metal complexes such as Prussian blue, metal-supramolecular polymers, or conductive organic polymers [20,21,22,23,24,25,26,27,28].

In the present work, we propose the implementation of an electrochromic device in a viologen/ferrocene-based system, with the dimensions of 10 cm × 10 cm, for future energy efficient smart windows. To make an ordered pattern of spaces, the photolithography technique has been applied. The cyclic voltammetry technique provides details of the redox process occurring inside the devices. Raman and UV-Vis-NIR spectroscopies have been used for the investigation of the electrochromism and the color switching of the devices as a function of the externally applied potentials by identifying the active species. The developed device shows its peculiar ability to modulate the radiation transmission in both visible and near-infrared regions, helping in the implementation of smart windows and improving the energy saving of the building.

## 2. Materials and Methods

### 2.1. Materials

Ethyl viologen diperchlorate ([EV(ClO_4_)_2_], 98% purity), 1,1′-diethyl ferrocene (DEFc, 98% purity), bisphenol-A glycerolate (1 glycerol/phenol) diacrylate (BPA, 98% purity), 2,2-dimethoxy-2-phenylacetophenone (Irgacure 651, 99% purity), propylene carbonate (PC) isopropanol (99.5% purity), and Sodium hydroxide (NaOH ≥ 97%, purity) were purchased from Sigma Aldrich Company (St Louis, MO, USA). Photoresist (SU-8 2010) and Developer (SU-8; 1-methoxy 2-propanol acetate) were supplied by MicroChem, Newton, MA, USA. A commercial masking film was provided by Fineline Imaging, Thin Metal Parts, Colorado Springs, CO, USA. All the chemicals have been stored in the refrigerator inside the clean room and have been used without any other form of purification.

### 2.2. Preparation of Electrochromic Gel

The electrochromic solution was made by adding the ethyl viologen diperchlorate and the diethyl ferrocene to the propylene carbonate with the following ratios in weight: 1.0, 0.6, and 14, respectively.

The polymeric component has been prepared by adding the photo-initiator, 2,2-Dimethoxy-2-phenylacetophenone (Irgacure 651) (2% *w*/*w*), to the Bisphenol-A glycerolate diacrylate (BPA). The mix has been subjected to slight heating at 100 °C on the plate in order to make the highly viscous BPA monomer more fluid and obtain, after stirring, homogenous polymeric mix.

The electrochromic gel was prepared by adding the electrochromic solution to the polymeric component in the following weight ratio: 40 and 60, respectively. Additionally, the electrochromic gel was slightly heated to obtain a homogeneous gel.

### 2.3. Fabrication of the Electrochromic Device

The electrochromic device is a multilayer system and can be schematized in the following way:

Glass substrate/ITO/electrochromic gel/ITO/Glass substrate

where the indium tin oxide (ITO) layer coated on the glasses are the two transparent electrodes where the external voltage is applied and that are divided by the electrochromic gel.

A photoresist was used for the realization of a tidy pattern of spacers with a thickness of 15–20 µm by the application of the photolithography technique following the process guidelines provided by Kayaku Advanced Materials, Inc. (Westborough, MA, USA) [29]. For the fabrication of the electrochromic devices, an accurate procedure was carried out in the clean room, where the yellow filter light was set in the room because, specifically for UV photosensitive applications, it blocks UV radiation reliably.

Previously all the ITO-coated glasses were cleaned with an aqueous solution of sodium hydroxide at 5% *w*/*w* followed by a deionized water rinse for the elimination of all traces of the cleaning solution. To dry off the glass, a jet of compressed air was used. The negative photoresist material SU-8-2010 was spin coated on the glass substrate obtaining a homogenous film of the desired thickness.

**Figure 1:**
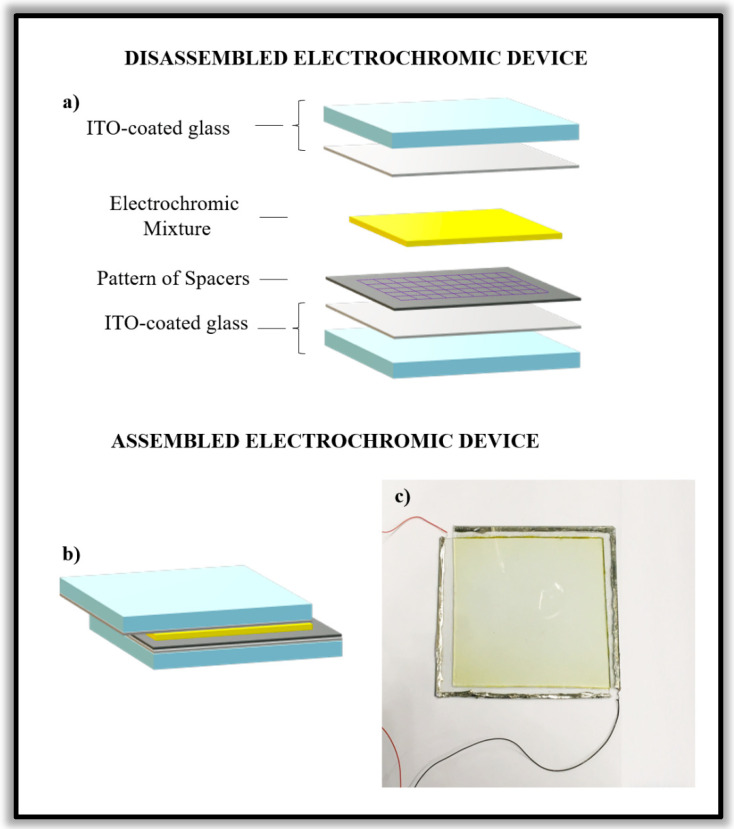
A schematic illustration of the electrochromic device in the different configurations: (**a**) the disassembled device; (**b**) the assembled device; and (**c**) a photograph of the obtained electrochromic device made with the electrochromic gel (described in the experimental section).

A soft bake step was needed to evaporate the solvent and densify the film. The resist-spin coated on an ITO-glass support was heated on a level hot plate at the temperature of 95 °C for a time of 3 min. For the exposing step, a mask for lithography and a sandwich configuration containing the sample was used. The photolithography mask is put on the resist coating. The system was closed by two clips. The exposing step was carried out under a UV lamp (350–400 nm) for a time of 2 min in a yellow-light condition. Following exposure, a Post Expose Bake (PEB) step of the sample was performed at the temperature of 95 °C for 4 min to selectively cross-link the exposed portions of the film. A controlled bake treatment is recommended to further cross-link the imaged resist structures. For the development step of the resist, the SU-8 developer was used under the operating chemical fume hood. The development of the film was performed with a multi-step bathing or immersion process in which the film was immersed in a developer solvent for 30 s. The substrate was rinsed briefly with isopropanol and then dried with a gentle stream of air. Assembly of the electrochromic devices with the surface area of 10 cm × 10 cm was performed. The electrochromic gel, described above, was put into the electrochromic device by the squeezing technique. A small amount of pressure was placed on the device to eliminate all excess electrochromic gel. The electrochromic device was sealed by epoxy glue and exposed to a UV lamp for 1 min to facilitate the polymerization of the BPA. For both the substrates of each electrochromic device, a tin wire of ITO was welded to the edges of the device and to electrical cables for the applied voltage. A schematic representation of the electrochromic device is given in Figure 1.

### 2.4. Characterization of the Electrochromic Device

Cyclic voltammetry measurements of the electrochromic devices were carried out in the cyclic potentials range from −2 V to 2 V at the scan rate of 50 mV/s using a Potentiostat/Galvonastat, model 2059 low instrument interfaced with Amel instruments—model 7800 interface (Amel Instruments, Perigny, France). Junior Assist software (2003) was used for the acquisition of the cyclic voltammetry measurement.

Raman spectra were acquired by using a Jobin Yvon micro-Raman LABRAM (Horiba Jobin Yvon, Piscataway, NJ, USA) equipped with a CCD detector cooled at −70 °C. An external laser source Nd:YAG (λ = 532 nm emission, 50 mW power) was used. A neutral filter of optical density (OD 0.3) was employed to change the laser power. A 50× MPlan Olympus with a numerical aperture of 0.75 was used, and the apparent diameter of the focused laser spot was about 2–3 µm. The spectral resolution was about 2 cm^−1^.

UV-Vis-NIR measurements were performed by the UV-Vis-NIR Spectrophotometer AvaSpec-2048-Avantes. The equipment included, a Deuterium-Halogen light source (DH-2000 UV-Vis-Nir Lightsource Avantes) and Agilent E3632A DC Power Supply (0–15 V, 7 A). Analyses were conducted at room temperature. Each UV-Vis-NIR measurement was conducted in the wavelength range 200–1200 nm. Avaspec75 software was used for the acquisition of UV-Vis-NIR measurements.

## 3. Results and Discussion

### 3.1. Cyclic Voltammetry

**Figure 2:**
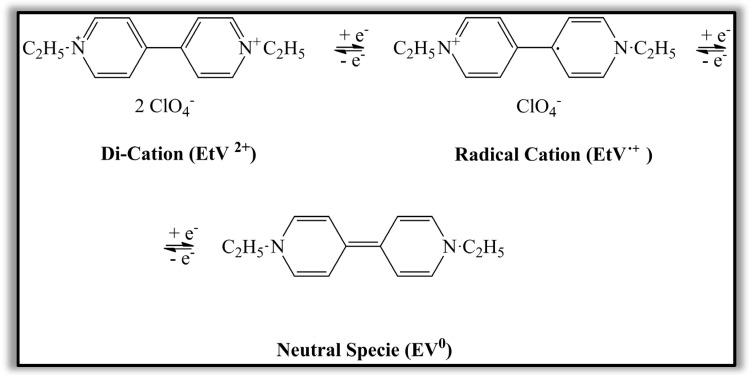
Schematization of the redox process occurring in the ethyl viologen diperchlorate under the application of a potential impressed on the electrode.

The investigated devices are based on the electrochromic gel containing the cathodic ethyl viologen diperchlorate, and the 1,1′-diethyl ferrocene as anodic species. As shown in Figure 2, in the presence of 1,1′-diethyl ferrocene, which is the reducing agent, the ethyl viologen diperchlorate can be reduced under the application of an electric field and becomes a radical cation.

Ethyl viologen diperchlorate (EV(ClO_4_)_2_) contains the EV^2+^ species representing a good electron acceptor, while the perchlorate ions act as centurions. Of course, the radical cations can be further subjected to a reduction process and become neutral species.

**Figure 3:**
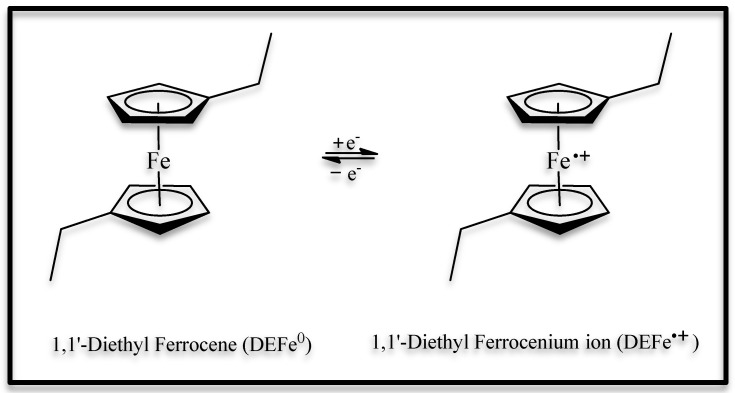
Schematization of the redox process that occurred in the 1,1′-Diethyl Ferrocene under the application of a potential impressed on the electrode.

Figure 3 shows the schematization of the redox process of the 1,1′-diethyl ferrocene when an external potential is applied to the electrochromic device.

The electrochemical properties of the electrochromic devices, obtained with the electrochromic gel described above, have been carried out by cyclic voltammetry. Cyclic voltammetry measurements were conducted in the potential range from 2 V to 2 V at the scan rate of 50 mV/s, and the electrochemical stability of the electrochromic devices has been tested by doing more than a hundred CV cycles.

**Figure 4:**
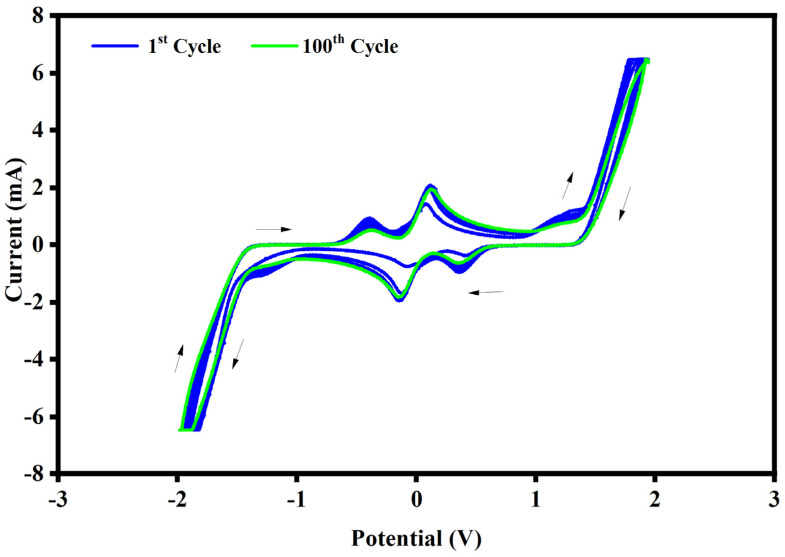
Cyclic voltammetry curve registered for electrochromic devices made with the electrochromic gel at the scan rate of 50 mV s ^−1^. The first cycle is represented by the green line while the other cycles, up to the 100th, are drawn by the blue line. The cathodic direction of the scanning of the potential is indicated by the black arrows.

The representative cyclic voltammetry curves (I–V) registered from the electrochromic devices are shown in Figure 4.

In the I-V curve, the cathodic current is imputable to two processes of the involved couples, at the same time: DEFc^0^/DEFc^•+^ and EV^2+^/EV^•+^. The first cathodic peak at about 0.35 V is ascribed to the oxidation of the DEFc^0^ to DEFc^•+^ species, while the second cathodic peak at about −0.14 V is referred to the reduction of EV^2+^ (di-radical cation) to EV^•+^ (cation radical). The two cathodic steps of the involved species can be individuated by the color changes of the electrochromic device. For one step, the reduction of the EV^2+^ to EV^•+^, the device shows a color variation from a light yellow to an intense dark blue color starting from about −1 V. The dark blue color of the electrochromic device is due to the presence of the radical cation EV^•+^. The I-V curve is symmetric, so this means that in the reversed scan, the anodic current is composed of two peaks, describing both the reduction of the DEFc^•+^ to DEFc^0^ at about −0.37 V and the oxidation of the EV^•+^ to EV^2+^ at about 0.13 V. As it is possible to see in Figure 4, the electrochromic performances of the devices do not change after 100 cycles: all the curves show the same peaks and are quite stacked.

**Figure 5:**
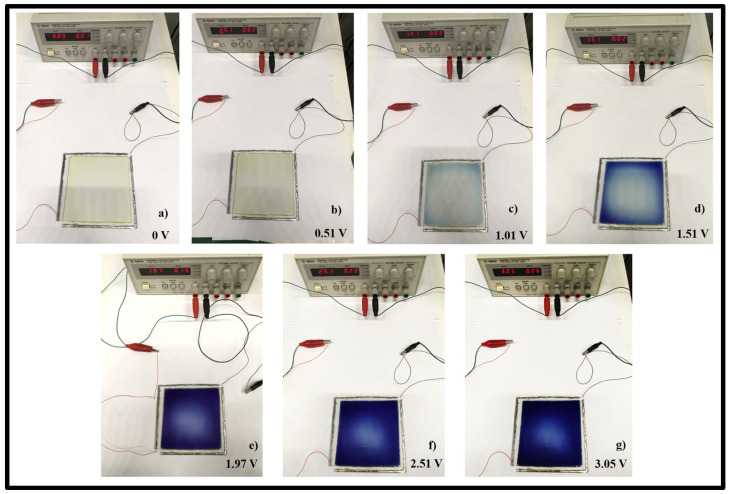
Electrochromic device based on the electrochromic gel in the OFF state at: (**a**) 0 V (light yellow coloration) and in the ON states at: (**b**) 0.5 V, (**c**) 1 V, (**d**) 1.5 V, (**e**) 2 V, (**f**) 2.5 V, and (**g**) 3 V (intense dark blue coloration).

The color variations recorded during the cyclic voltammetry experiments are shown in Figure 5. When the external potential is equal to 0 V, the electrochromic device is in the transparent state (light yellow coloration), while the dark blue color is originated by the presence of reduced species of the viologen starting at about 1 V up to 3 V.

The reason for the uneven color change of the electrochromic devices shown in Figure 5, is ascribed to the electrical potential drop of the potential applied, which is responsible for a lower applied voltage at the center of the device with respect to that applied on the borders of the devices. Increasing the duration of the application of the voltage allows for obtaining a uniform color change.

### 3.2. Micro-Raman Spectroscopy

Raman spectroscopy has been widely used for studying many kinds of systems, such as: semiconductors, polymeric gel, phthalocyanine films, carbon-based systems (graphene and nanotubes), biopolymers, biomimetic biological systems and so on [30,31,32,33,34,35,36,37,38,39,40,41,42,43].

In this work, Raman measurements have been conducted for studying the redox process that occurred to the EV^2+^/EV^•+^ couple in the electrochromic device, which is responsible for the color variation of the device.

**Figure 6:**
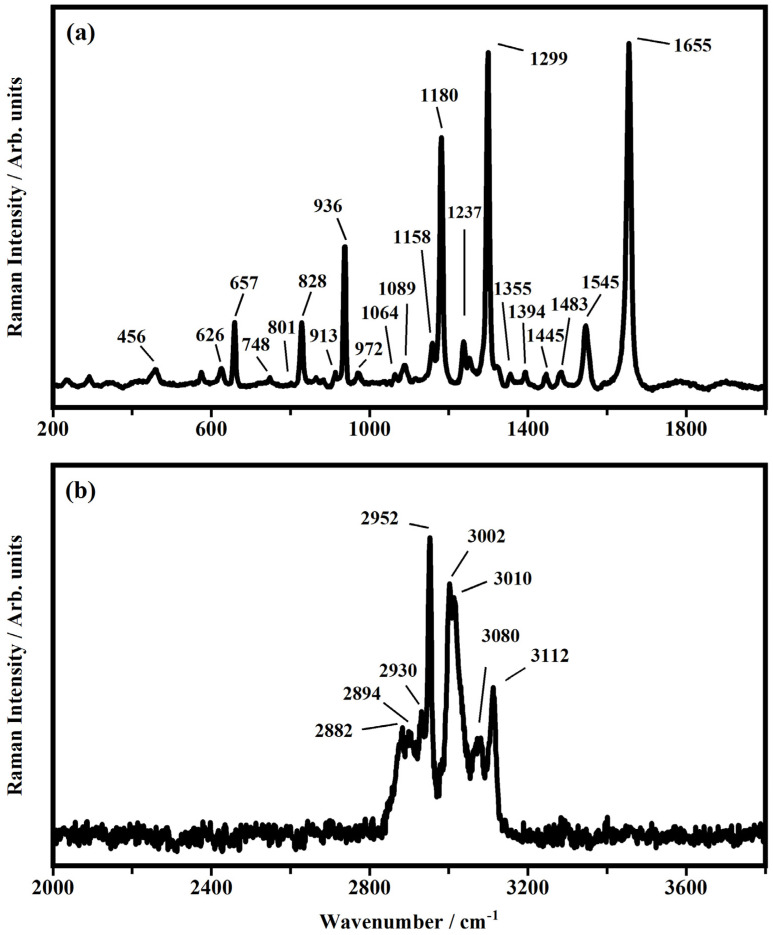
Raman spectra of ethyl viologen diperchlorate EV(ClO_4_)_2_ powder collected in ranges (**a**) from 200 to 2000 cm^−1^ and (**b**) from 2000 to 3800 cm^−1^.

First of all, the ethyl viologen diperchlorate powder, EV(ClO_4_)_2_ has been used as a starting reference, and a tentative attribution of its vibrational modes has been conducted in accordance with the literature [17,44,45,46,47,48,49,50,51,52]. The representative Raman spectra collected on the powder of EV(ClO_4_)_2_ are shown in Figure 6. The vibrational bands located at about 456 and 626 cm^−1^ are assigned to the asymmetric and symmetric bending of the perchlorate anions. The stretching of the N-CH_2_CH_3_ bond is found at 657 cm^−1^. The band observed at 748 cm^−1^ is related to the out-of-plane vibration of the C-H bond. At 801 cm^−1^ and at 828 cm^−1^ the stretching of the C-N bond and the stretching of the C-C and N-H bond of the pyridine group in the viologen occurred. The vibrational bands located at 913 and 936 cm^−1^ are assigned to the symmetric stretching of the Cl-O bond in the diperchlorate anion. At 972 cm^−1^ the out-of-plane ring vibration of the pyridine is found. The bands at 1064 cm^−1^ and 1180 cm^−1^ reflect the ring breathing vibrations in the pyridine. The asymmetric stretching of the Cl-O bond of the diperchlorate anion is observed at 1089 cm^−1^. The in-plane ring bending mode of the bond H-C-C is at 1237 cm^−1^.

The in-plane ring bending of the ring is found at 1248 cm^−1^. The band at 1299 cm^−1^ describes the inter-ring vibration of the C-C bond and the bending inter-ring vibration of the H-C-C bonds. At 1355 cm^−1^ the C-C inter-ring vibration occurs, and at 1394 cm^−1^ the out-of-plane bending of CH_3_ groups occurs, while the band at 1445 cm^−1^ describes the asymmetric C-H bending vibration. The deformation of the C-H bonds is found at about 1483 cm^−1^ [50].

The bending vibration of the H-C-C bond and the stretching of the C-N bond occurred at 1545 cm^−1^. The C-C inner-ring vibrations of the ring are found at 1655 cm^−1^. In the region between 2800 cm^−1^ and 3100 cm^−1^ the symmetric and the asymmetric stretching vibrations of the C-H bonds are located. The symmetric and asymmetric vibrational stretching of the C-H bond occurred at 2882 cm^−1^ and at 2952 cm^−1^. The bands at 2894 cm^−1^ and 2930 cm^−1^ describe the asymmetric and the symmetric stretching of the methylene group. The stretching of the C-H bond in the methyl group is found at 3002 cm^−1^. The band at 3010 cm^−1^ is due to a combination of the bands at 1655 cm^−1^ and 1355 cm^−1^. Bands located at 3080 cm^−1^ and 3112 cm^−1^ are referred to by the stretching of the aromatic C-H bond. In Table 1 the main vibrational assignments collected on the ethyl viologen diperchlorate powder as EV^2+^ species are summarized.

**Figure 7:**
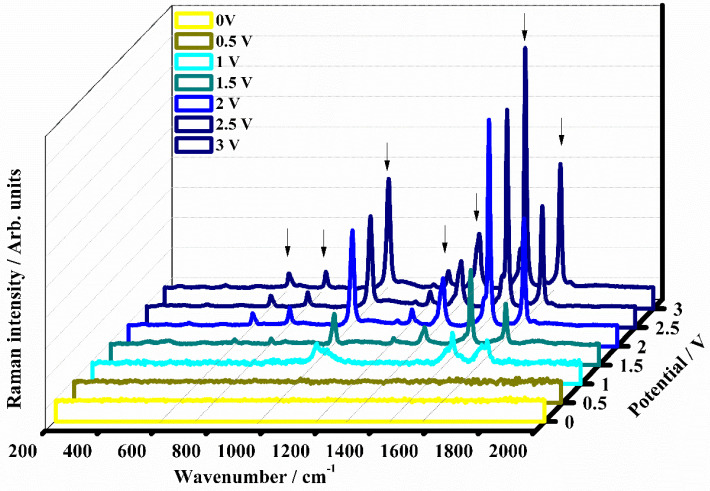
Raman spectra of the electrochromic device based on the concentration (60:40) % *w*/*w* based on (EV(ClO_4_)_2_:DEFc:PC)-(BPA-Irgacure 651) collected in the range from 200 to 2000 cm^−1^ as a function of the external applied potential: 0 V (OFF state, yellow line), 0.5 V (dark yellow line), 1 V (ON state, cyan line), 1.5 V (dark cyan line), 2 V (blue line), 2.5 V (navy blue line), and 3 V (royal blue line).

Following the structural evolution of the ethyl viologen diperchlorate associated with the redox process, as a function of the applied external potential (from 0 V to 3 V), the Raman spectra of the electrochromic device have been collected in the range between 200 cm^−1^ and 2000 cm^−1^, and they are shown in Figure 7.

A tentative attribution of the main vibrational modes is presented in accordance with the literature [24,47,53,54]. The Raman spectra collected on the electrochromic device under 0 V and 0.5 V species do not show appreciable Raman signals, because they are masked by strong fluorescence and the laser wavelength excitation is not sufficient to induce an appreciable chemical bond resonance.

The Raman spectra collected in the potential range from 1 V to 3 V, are characterized by the appearance of new bands that can be attributed to the stable radical cation of the ethyl viologen diperchlorate (EV^•+^). At 1 V, the electrochromic device shows a first appreciable color variation, which seems to be light blue. This light color variation corresponds to the starting of the reduction of the EV^2+^ species to the EV^•+^ species. Characteristic bands associated with the reduced EV^•+^ species have been observed in the Raman spectrum. The first band is located at 1030 cm^−1^ and it is ascribable to the ring breathing vibration of the C-C bond. The second band is found at 1528 cm^−1^ and it is due to the bending vibration of the C-H bond and to the ring vibration of the C-H bond. The band at 1640 cm^−1^ is associated with the EV^2+^ species, the band at 1655 cm^−1^ is assigned to the EV^•+^ species, and both are assigned to the ring vibration of the C-C bond. The coexistence of both vibrational bands represents clear evidence that the reduction process of the ethyl viologen diperchlorate is taking place. The observation of all these mentioned bands confirms the accomplished reduction of the ethyl viologen diperchlorate as a di-cation species EV^2+^ in the radical cation EV^•+^, as is also justified by the starting color variation of the electrochromic device. The increasing of the potential, leads to obtaining a more detailed Raman spectrum of the reduced species. At 1.5 V the Raman spectrum is characterized by the appearance of bands that are located at 661, 797, 1027, 1246, 1360, 1529 cm^−1^, and 1658 cm^−1^.

The bending modes of the C-N-C and C-C-N bonds are located at 661 cm^−1^. The band at 797 cm^−1^ is due to the ring stretching vibration of the C-N bond. The ring breathing vibration of the C-C bond is confirmed at 1027 cm^−1^. The in-plane bending vibration of the H-C-C bond is found at 1246 cm^−1^, while at 1360 cm^−1^ the inter-ring vibration of the C-C bond is observed. At 1529 cm^−1^ the bending and the ring vibration of the C-H bond can be observed. The band located at 1658 cm^−1^ is assigned to the C-H ring vibration. The Raman spectrum collected under the potential of 2 V confirmed the bands located at 661, 797, 1246, 1529, and 1658 cm^−1^, previously assigned to the reduced species of the ethyl viologen diperchlorate. In addition, a new band at about 1194 cm^−1^ concerning the vibration of the N-(CH_2_) bond is revealed. In the Raman spectrum collected at 2.5 V, the bands ascribed to the radical cation EV^•+^ have been confirmed at 661, 797, 1028, 1194, 1246, 1361, 1529, and 1658 cm^−1^. When the external potential is set to the value of 3 V, the color variation from light blue to the intense dark blue color is more appreciable. The device exhibits a dark blue color, confirming the presence of the radical cation EV^•+^. The representative Raman spectrum is well-defined and confirms the bands at 661, 797, 1028, 1192, 1247, 1362, 1529 and, 1658 cm^−1^.

**Figure 8:**
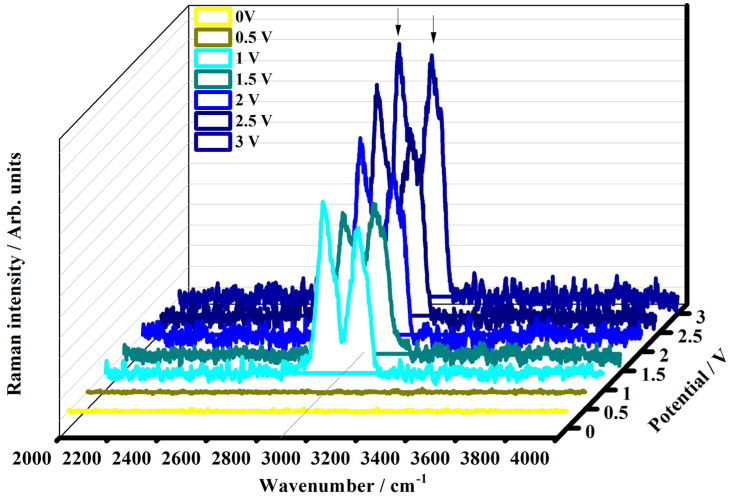
Raman spectra of the electrochromic device made by the electrochromic gel collected in the range of 2000–4000 cm^−1^ as a function of the external applied voltage: 0 V (OFF state, yellow line), 0.5 V (dark yellow line), 1 V (ON state, cyan line), 1.5 V (dark cyan line), 2 V (blue line), 2.5 V (navy blue line), and 3 V (royal blue line).

Figure 8 shows the Raman spectra collected on the electrochromic device under the application of the potential from 0 V to 3 V in the range between 2000 and 4000 cm^−1^.

In the Raman spectra collected at 0 V, and 0.5 V no detectable Raman signals can be observed. The Raman spectra collected on the device in the potential range from 1 V to 3 V are characterized by the presence of some bands located between 2880 and 3050 cm^−1^. The stretching vibration of the C-H bond is found at 2882 cm^−1^ in the spectra collected at 1 V, 2 V, and 3 V and at 2880 cm^−1^ in the spectra collected at 2.5 V. The band at about 3015 cm^−1^ is due to the combination of the bands located at about 1655 cm^−1^ and 1355 cm^−1^. The weak band at 3050 cm^−1^ (1.5 V), 3054 cm^−1^ (2.0 V), 3050 cm^−1^ (2.5 V), and 3051 cm^−1^ (3.0 V) is the overtone of the band at about 1528–1529 cm^−1^.

In Table 2 the main vibrational assignments of the reduced EV^•+^ species observed in the ranges of 200–2000 cm^−1^ and 2000–4000 cm^−1^ from 0 V to 3 V are summarized.

### 3.3. UV-Vis-NIR Spectroscopy

**Figure 9:**
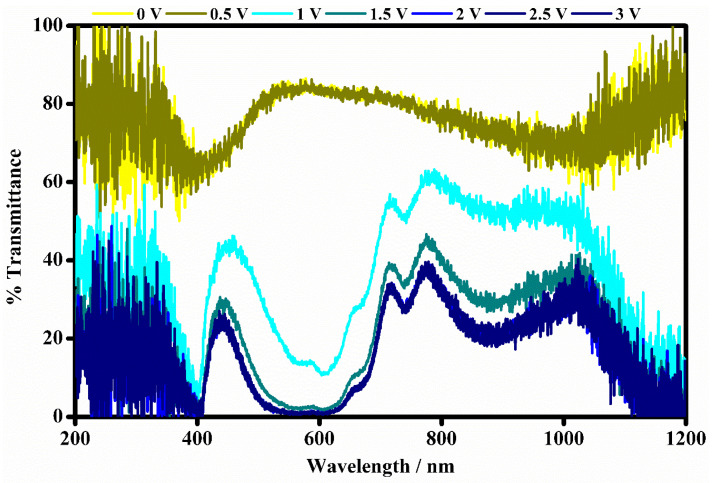
The optical transmittance spectra of the fabricated device at different potentials ranging from 0 V to 3 V registered between 200 and 1200 nm wavelengths.

UV-Visible-NIR spectroscopy has been used for the optical characterization of the electrochromic device based on the (60:40)% *w*/*w* (EV(ClO_4_)_2_):(DEFc):(PC) and (BPA-Irgacure 651) mixture. The transmittance of the electrochromic device has been measured in the range from 200 to 1200 nm, during the gradual increasie of the applied voltage in the range from 0 V to 3 V. In Figure 9, the transmission spectra are shown.

The electrochromic device shows interesting transmittance modulation as a consequence of the presence of the EV^2+^/EV^•+^ couple. At 0 V the electrochromic device appears in its OFF state. It transmits all wavelengths of the UV-Vis-NIR region of the electromagnetic spectrum except at about 560 nm where there is a peak assigned to the EV^2+^ species and thus appears a light yellow, transparent color. Then, as the potential is increased from 0.5 V to 3 V (in steps of 0.5 V), the electrochromic device is in its ON state which is associated with the color variation of the electrochromic device. In fact, the blue color of the device is directly observable by the naked eye, and it is ascribed to the reduced EV^•+^ species. The peak at 560 nm is suppressed starting from the applied voltage of 1 V, and a clear change in transmission is visible both in the visible region at about 400 nm 606 nm wavelengths and in the NIR region at about 890 nm wavelength.

The transmittance modulation (%) of the electrochromic device has been evaluated when the device is put in two operative conditions: the bleached state at 0 V and the colored state at 1 V and 3 V.

**Figure 10:**
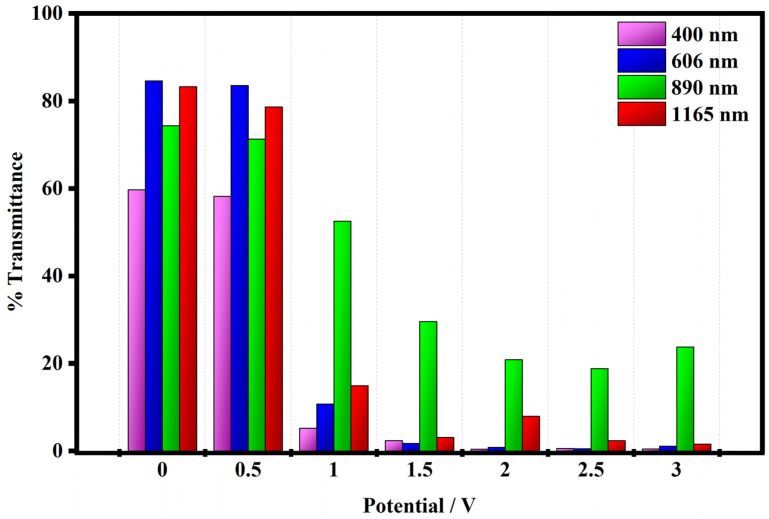
The transmittance modulation of the fabricated device at 400 nm, 606 nm, 890 nm, and 1165 nm wavelengths registered at 0 V, 1 V, and 3 V.

The trends of the variation of the transmittance modulation are plotted in Figure 10 and have been evaluated at maximum contrast wavelength (400 nm, 606 nm, 890 nm, and 1165 nm) i.e., where the device exhibited minimum transmittance in the UV-Vis-NIR region.

As shown in Figure 10 the electrochromic device shows interesting transmittance modulations in the wide range from 200 to 1200 nm. In the visible range at 400 nm, the device shows a transmittance of 59.6% at 0.0 V (OFF state, bleached state). At 1 V, when the device undergoes the redox process, the value of the transmittance decreases to 5.14%, and at the maximum color variation at 3 V its transmittance is equal to 0.48%. At 606 nm, the electrochromic device exhibits a transmittance of 84.6% under 0 V, and it is fully bleached. Then, the transmittance is drastically reduced to 10.71% at 1 V which corresponds to the first color variation when the system is put in the ON state and then to 1.10% at 3 V where the system is fully blue colored.

In the near-infrared wavelength region (NIR), the transmittance modulations of the device have been evaluated at 890 and 1165 nm wavelengths. At 890 nm the device shows transmittance of 74.3% in the bleached state (0 V), while in the ON states at 1 V the transmittance is reduced to 52.5% and at 3 V it is equal to 23.7%. At 1165 nm, the transmittances of the device change from an initial value of 83.2% in the OFF state to values of 14.9% at 1 V and 1.58% at 3 V corresponding to the ON states of the device.

Furthermore, some optical properties such as the color contrast ratio, (CCR (%)) and the coloration efficiency (CE) have been calculated between the OFF state (0 V) and ON state (3 V) when the device is fully colored.

The color contrast ratio, *CCR* (%) is defined as the contrast between bleached and colored states as follows (Equation (1)) [5]:

(1) $$CCR\;\left(\%\right)=\frac{T_{bleached}-T_{colored}}{T_{bleached}}\times 100$$

where *T_bleached_* and *T_colored_* are the bleached/initial and the colored/final transmittance values at 400 nm, 606 nm, 890 nm, and 1165 nm wavelengths.

The performances of the electrochromic device are interesting in both the complete visible range and in the near-infrared region. The device exhibits higher contrast color ratios at 400 nm and 606 nm with values of 99.1% and 98.7%, while in the near-infrared region, at 890 nm and 1165 nm, the values of color contrast range are equal to 68% and 98.1%.

To compare the performances of the electrochromic devices, another parameter such as the coloration efficiency can be taking into account. The coloration efficiency is defined as the change in optical density (Δ*OD*) at a particular wavelength (*λ*) per unit area of charge (*Q*) intercalated or extracted from the electrochromic film as follows (Equation (2)) [5]:

(2) $$CE\left(\lambda \right)=\frac{∆OD\left(\lambda \right)}{Q}=\frac{\log\left(\frac{T_{b}}{T_{c}}\right)}{Q}$$

where $T_{b}$ and $T_{c}$ represent the transmittance in bleached and colored states at 400 nm, 606 nm, 890 nm, and 1165 nm wavelengths.

The electrochromic device promotes higher values of coloration efficiency in the investigated wavelength range. In the visible range, at 400 nm, the value of CE is equal to 510.6 cm^2^/C and at 606 nm is 460.3 cm^2^/C. In the NIR region, the coloration efficiency values are equal to 120.9 cm^2^/C at 890 nm and 420.1 cm^2^/C at 1165 nm. These results seem to be promoting this model of reducing the energy consumption of buildings compared to the commonly used electrochromic, viologen-based devices reported in the literature [55,56,57,58].

In Table 3 all optical data estimated for the electrochromic device are summarized.

## 4. Conclusions

An organic electrochromic device has been made based on the gel made by a mixture of ethyl viologen diperchlorate, 1,1′-Diethyl Ferrocene, Bisphenol-A glycerolate diacrylate, and Irgacure 651. The electrochromic device shows a color change from transparent at 0 V (OFF State) to the intense dark blue color (ON State) starting from 1 V to 3 V, which is associated with the redox process of the EV^2+^/EV^•+^ couple.

Cyclic voltammetry measurements confirmed the reversible behavior of the device under the application of an external voltage.

Micro-Raman spectroscopy has been conducted in the applied voltage range between 0 V and 3 V and confirmed the presence of the EV^•+^ as justified by the blue color of the electrochromic device in the ON state.

The electrochromic devices exhibit high transmittance modulations and coloration efficiencies, thus making themselves suitable devices for the fabrication of smart windows able to modulate the indoor lighting in both visible and near-infrared regions and improving the energy saving of the buildings.

UV-Vis-NIR spectroscopy confirmed the transmittance modulation of the device in the visible region and in the near-infrared region. At 400 nm the transmittance of the device varies from 59.68% (OFF state, at 0 V) to 0.48% (ON state, 3 V) and at 606 nm from 84.58% (OFF state, 0 V) to 1.01% (ON state, 3 V). In the NIR region, at 890 nm the device shows a transmittance of 74.33% (OFF state, 0 V) and a decrease of up to 23.76% (ON state, 3 V), while at 1165 nm the values of the transmittance changed from 83.21% in the OFF state to 1.58% in the ON state at 3.0 V.

The electrochromic device shows high values of CCR% in both visible and near-infrared regions when it switched between OFF/ON states. At 400 nm it is characterized by a CCR% of 99.18%, at 606 nm it shows a CCR% of 98.69, and a CCR% of 68.02% at 890 nm. Furthermore, it exhibits excellent values of CE in both visible and NIR regions. At 400 nm we obtained a CE of 510.6 cm^2^/C, while at 606 nm the device shows a CE of 460.3 cm^2^/C, which is suitable for the realization of smart windows with high coloration efficiencies. In the NIR region at 890 nm, it can be used for energy-saving in buildings with a promising CE of 120.9 cm^2^/C and 420.1 cm^2^/C at 1165 nm.

## Figures and Tables

**Table 1:** Main vibrational assignments of the diethyl viologen diperchlorate powder.

Raman Shift (cm^−1^)	Assignments	Raman Shift (cm^−1^)	Assignments
456	ClO_4_^−^ symmetric bending	1299	C-C inter-ring vibration + H-C-C inter-ring bending
626	ClO_4_^−^ asymmetric bending	1355	C-C inter-ring vibration
657	N-CH_2_CH_3_ stretching	1394	CH_3_ out-of-plane-bending
748	C-H out-of-plane vibration	1445	C-H asymmetric bending
801	C-N ring stretching	1483	C-H vibration
828	C-C and -N-H stretching (pyridine group)	1545	H-C-C bending + C-N stretching
913	Cl-O symmetric stretching	1655	C-C inner ring vibration
936	Cl-O symmetric stretching	2882	C-H asymmetric stretching
972	Out-of-plane of ring	2894	CH_2_ asymmetric stretching
1064	C-N and C-C stretching (ring breathing vibration)/asymmetric stretching Cl-O)	2930	C-H symmetric stretching of CH_2_
1089	Cl-O asymmetric stretching	2952	C-H symmetric stretching
1158	Cl-O asymmetric stretching	3002	C-H stretching of CH_3_
1180	N-CH_2_CH_3_ stretching	3010	Combination of 1655 cm^−1^ + 1355 cm^−1^
1237	H-C-C in-plane bending	3080	Aromatic C-H stretching
1248	H-C-C in-plane bending of the ring	3112	Aromatic C-H stretching

**Table 2:** Main vibrational assignments of the diethyl viologen diperchlorate present as the radical form of EV^•+^ in the electrochromic device in the range of applied voltage between 1 V and 3 V in the ranges between 200 and 2000 cm^−1^ and between 2000 and 4000 cm^−1^.

Appied Voltage/V							Assignments
**0**	0.5	1	1.5	2	2.5	3	
Peak Position/cm^−1^							
**/**	/	/	661	661	661	661	C-N-C bending + C-C-N bending
**/**	/	/	797	797	797	797	C-N ring stretching
**/**	/	1030	1027	1028	1028	1028	C-C ring breathing
**/**	/	/	/	1194	1194	1192	N-(CH_2_) vibration
**/**	/	/	1246	1246	1246	1247	H-C-C in-plane bending vibration
**/**	/	/	1360	1361	1361	1362	C-C inter ring vibration
**/**	/	1528	1529	1529	1529	1529	C-H bending + C-H ring vibration
**/**	/	1640/1655	1658	1658	1658	1658	C-C ring vibration
**/**	/	2882	2882	2882	2880	2882	C-H asymmetric stretching
**/**	/	3010	3011	3010	3011	3011	Combination of 1655 cm^−1^ + 1355 cm^−1^
**/**	/	/	3050	3054	3050	3051	Overtone of 2 × 1528 cm^−1^ (1529 cm^−1^)
**/**	/	2882	2882	2882	2880	2882	C-N-C bending + C-C-N bending
**/**	/	3010	3011	3010	3011	3011	C-N ring stretching
**/**	/	/	3050	3054	3050	3051	Overtone of 2 × 1528 cm^−1^ (1529 cm^−1^)

**Table 3:** Transmittances of the OFF state (0 V) and ON states (1 V, and 3 V), color contrast ratio (CCR%), coloration efficiency values in the visible region (λ = 400, 606 nm) and near infrared region (λ = 890 nm, 1165 nm) for the electrochromic device made by the electrochromic gel.

λ (nm)	Q/Surface (C/cm^2^) (abs. Values)	Transmittance (%)							CCR% at 3 V (%)	CE at 3 V (cm^2^/C)
		**0.0 V**	**0.5 V**	**1.0 V**	**1.5 V**	**2.0 V**	**2.5 V**	**3.0 V**		
**400**	4.09 × 10^−^^3^	59.68	58.20	5.14	2.34	0.40	0.54	0.48	99.18	510.6
**606**	4.09 × 10^−^^3^	84.58	83.52	10.71	1.74	0.84	0.53	1.10	98.69	460.3
**890**	4.09 × 10^−^^3^	74.33	71.28	52.51	29.56	20.81	18.83	23.76	68.02	120.9
**1165**	4.09 × 10^−^^3^	83.21	78.66	14.91	3.10	1.94	2.34	1.58	98.0	420.1

## Data Availability

The data presented in this study are available upon request from the corresponding author.
